# Selection of Reference Genes for Real-Time Quantitative PCR in *Pinus massoniana* Post Nematode Inoculation

**DOI:** 10.1371/journal.pone.0147224

**Published:** 2016-01-22

**Authors:** Yongcheng Wei, Qinghua Liu, Hongyu Dong, Zhichun Zhou, Yanping Hao, Xuelian Chen, Liuyi Xu

**Affiliations:** 1 Research Institute of Subtropical Forestry, Chinese Academy of Forestry, Fuyang, Zhejiang 311400, China; 2 Zhejiang Provincial Key Laboratory of Tree Breeding, Fuyang, Zhejiang, 311400, China; 3 State Forestry Administration Engineering Research Center of Masson Pine, Fuyang, Zhejiang 311400, China; 4 Anhui Academy of Forestry, Hefei, Anhui 230000, China; Northwestern University, UNITED STATES

## Abstract

*Pinus massoniaia* Lamb has gained more and more attention as the most important tree species for timber and forestation in South China. Gene expression studies are of great importance to identify new and elite cultivars. Real-time quantitative PCR, a highly sensitive and specific method, is commonly used in the analysis of gene expression. The appropriate reference genes must be employed to normalize the calculation program for ascertaining repeatable and significant results. Herein, eleven housekeeping genes were evaluated during different stages of *P*. *massoniana* post nematode inoculation in this study. Three statistical approaches such as geNorm, NormFinder and BestKeeper were selected to analyze the stability of candidate genes. The results indicated that *U2af* and *β-TUB* were the most stable reference genes. These two genes could be used for the normalization in most of the experiments of *P*. *massoniana*, while *Histone* and *AK* were the least stable ones. In addition, *EF* expressed at the lowest average Ct value was the most abundant candidate gene. As an important gene associated with defense mechanisms, ABC transporter was analyzed by qRT-PCR, and the results were used to confirm the reliability of two genes. The selected reference genes in the present study will be conducive to future gene expression normalized by qRT-PCR in *P*. *massoniana*.

## Introduction

Pine wilt disease (PWD) caused by pine wood nematode (PWN), *Bursaphelenchus xylophilus*, is believed to be the most destructive disease of pine species [1–3], causing significant economic and ecological losses around the world. Totally 40 000 000 m^3^ pine forests were damaged by PWD in Japan[4] and PWN has been spread in 16 provinces in China[5]. Additionally, the pathogenic mechanism of PWN has been reported in relation to the dynamic variation process of cell morphology, physiological and biochemical progress in pines based on different studies[6–13].Recently, selecting PWD-resistant varieties through breeding program has been considered as an efficient means to reduce the damage of pine forest by PWN, and many successful transplantation cases have been reported [14]. However, the resistance mechanisms of selected varieties have not been gained enough attentions although *Pinus massoniana* is one of the most important tree species for timber and forestation in South China, and its high susceptibility to PWN and resultant economic loss have also been reported [15]. In the past, traditional strategies are focused on choosing the survival clones from generation to generation under artificial infection. Therefore, these studies have provided excellent experimental materials such as, clones, varieties or elite lines for following research [14]. However, the underlying molecular mechanisms in the resistant or susceptible trees after infection have not been reported. We conducted a transcriptomics study to identify differentially expressed genes in resistant and susceptible clones. However, it is necessary to validate the change in gene expression by quantitative real-time PCR (qRT-PCR), which needs more accurate reference genes with stable expression in all stages post inoculation to normalize the internal expression. In the previous study, the reference genes commonly used in pine trees were *actin*, *elongation factor*, *tubulin*, *adenosine kinase* and *clathrin adaptor complex subunit* [5, 16–19].

The qRT-PCR has become one of the most effective approaches to analyze gene expression accurately for many biological systems and various experimental treatments due to its low template input, high sensitivity, and high specificity for the quantitative detection of delicate diversities in different samples [16, 18, 20–23]. Meanwhile, it is a time-saving, cost-effective and widely-used approach when compared to microarray and Northern blotting [17, 19, 24–25]. However, the arguable imperfection of qRT-PCR is the variation arising not only from subsistent biodiversity but also from operational factor, which will be the cause of the nonspecific variation [20, 26]. The variation from initial quantification must be reduced by normalizing Ct values during different experimental treatments [25]. Some approaches reported by Thellin et al. [27] for relative normalization have demonstrated that the first choice is to choose ideal reference genes for maintaining expression stability against internal and external variation from experimental treatments and target genes. Meanwhile, many researchers suggest that the ideal reference gene can provide more convincing results when several endogenous genes are employed in the same detecting system [28], since all studies have not revealed a single gene for all samples and the results may be influenced by continually employed reference genes under certain treatments [29–31]. According to Vega-Bartol’s report[19], the stability and abundance of reference genes can influence the accuracy of normalization, indicating that the necessity for estimating stability of reference genes should be prior to normalizing target gene reactions. Therefore, the evaluation of feasible reference gene’s stability should be conducted in the operational conditions before normalization during quantification [32]. Several studies have confirmed the importance of reference genes with or without stability in quantitative analysis [19, 33–36]. geNorm [32], NormFinder [37] and BestKeeper [38] are the most commonly used methods to evaluate reference genes. Different statistical algorithms can result in inconsistent ranking. Given different conditions and statistical algorithms, the candidate genes with stable expression can be the reference genes for the normalization of qRT-PCR results [32, 39–40].

It is presumed that some differentially expressed genes between resistant and susceptible clones of *P*. *massoniana* may be the crucial factor against PWN. The selection of reference genes should be prior to the quantification of these genes. In this study, we exploited the transcriptome data of *P*. *massoniana* and picea (*Picea asperata* Mast.) to search the candidate reference genes. Eleven housekeeping genes commonly involved in gene expression were tested, and the stability in resistant and susceptible clones of *P*. *massoniana* during three stages post inoculation was analyzed. Our results identified the appropriate reference genes with stable expression both in different resistant clones and in three phases of the samples. The selected reference genes were validated by normalizing the target gene associated with defense mechanisms, and the significant difference between resistant and susceptible clones at different stages was explored. This work has provided the suitable reference genes for the normalization of qRT-PCR to analyze the expression of target genes accurately in clones of *P*. *massoniana*.

## Results

### Identification of candidate reference genes and primer specificity and efficiency

**Fig 1:**
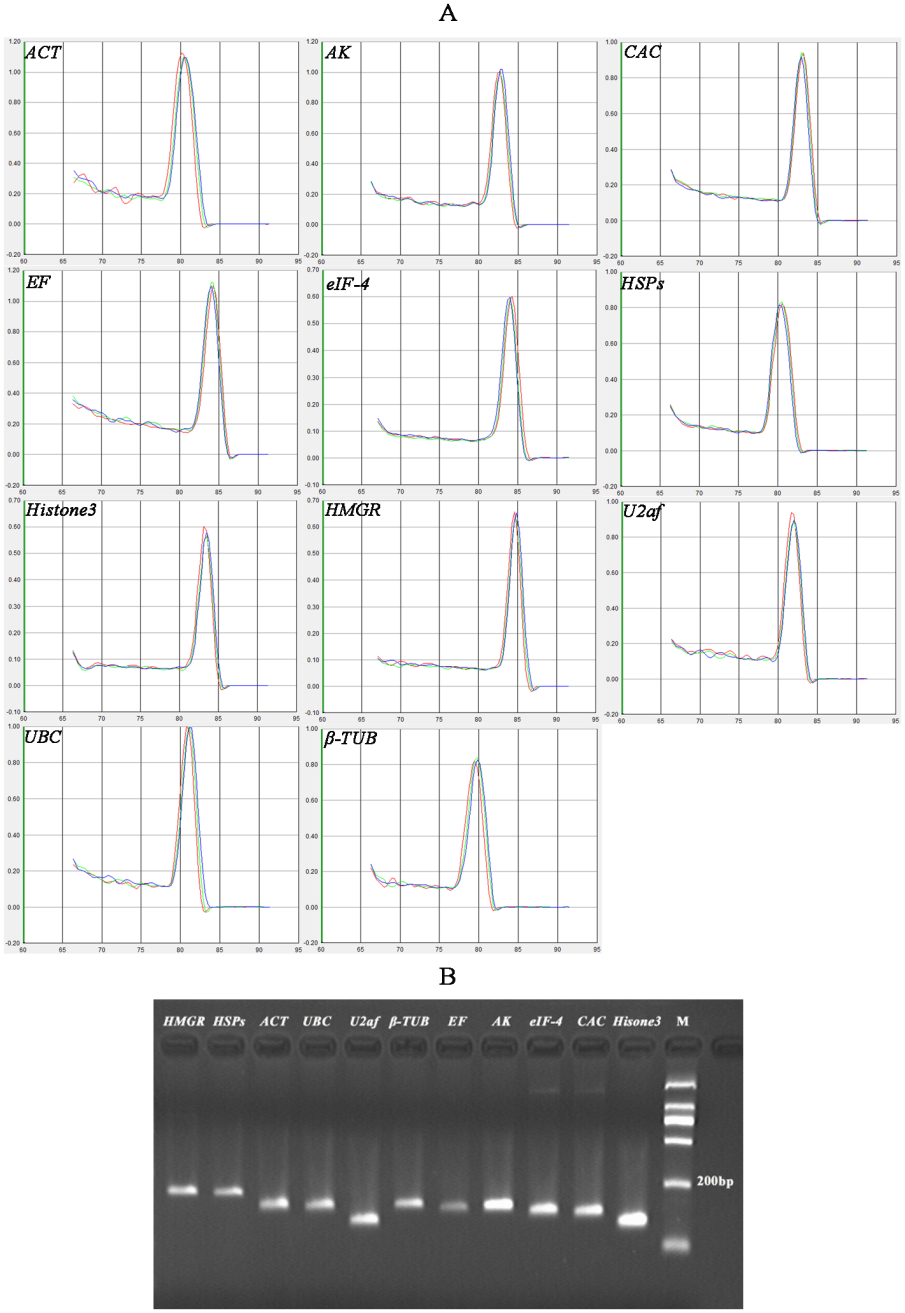
Specificity of RT-qPCR amplification. A) Melting curve of the 11 reference genes showing a single pick (each including three technical replicates of the cDNA pool of the total samples used in this study). B) Agarose gel (2%) showing amplification of a specific PCR product of the expected size for each gene tested in this study.

According to previous studies, eleven genes were selected as the candidates for the normalization of qRT-PCR through searching the reference genes from orthologs of *Picea* and *Pinus* in susceptible and resistant *P*. *massoniana* transcriptome database. These genes include *actin* (*ACT*), *adenosine kinase* (*AK*), *clathrin adaptor complex subunit* (*CAC*), *elongation factor* (*EF*), *eukaryotic initiation factor 4α*(*eIF-4*), *heat shock protein(HSPs)*, *histone3* (*Histone3*), *3-hydroxy-3-methyl glutarylcoenzyme A reductase* (*HMGR*), *splicing factor* (*U2af*), *ubiquitin conjugating enzyme* (*UBC*), and *tublin* (*β-TUB*) (Table 1). These gene products are associated with a variety of key biological functions. A subsistent single peak indicates that the qRT-PCR product is specific. The expected size of the products with a single fragment varied from 120 to 200 bpin2% agarose gel electrophoresis and gel red nucleic acid staining confirmed the amplification specificity (Fig 1). The estimated PCR amplification efficiency of these reference genes ranged from 1.464(*AK*) to 1.590(*ACT*), and the correlation coefficients (*R*^*2*^) varied between 0.9963(*HMGR*) and 0.9984(*EF*), respectively.

**Table 1:** Primer sequences and amplification of 11 candidate reference genes evaluated in this study.

*Genes*	Primer sequences (forward/reverse)	Amplification length(bp)	Amplification efficiency	R^2^
*ACT*	CCAAGGCAAACAGAGAGAGGA/GTCCAGATTCAAGCGGACCT	189	1.59	0.9967
*AK*	TCTCTGCCCCAAACTTGTCC/CAACGCGATATTGGCAGAGC	185	1.464	0.9981
*CAC*	TACTGCTAACACGGTTGCGT/TGCTCAGAACCACCGACAAT	159	1.51	0.9975
*EF*	AGCGTGAAGTGTGACATCGT/GTCCAGATTCAAGCGGACCT	176	1.568	0.9984
*eIF-4*	CTGAAGCCCCATGGAGTCAA/GCAGATCAAGGGTTTCTCAGC	178	1.491	0.9971
*HSPs*	AAAGAGCACCGGTGGAAGAG/TTTCCTCGTGCGGCATATCA	194	1.479	0.9969
*Histone3*	TGTGGTTTGTGTGGGGTTGG/CCAATCCACAGCCCTCGAAT	120	1.528	0.9968
*HMGR*	GGCGATCCCAAAGAATCCCA/TCTGCCTCTTCCCGTTACCT	196	1.562	0.9963
*U2af*	TCGGGAGGTTGGGTCTACAT/ACCAGTCCTTCAGTCCCCTT	140	1.487	0.9971
*UBC*	AGGAGTATCAGCAGGACCCA/TTTGGGTTGGCGCTGAATTG	187	1.494	0.9979
*β-TUB*	GTCGTGAATCATGGCATGGC/GCCTCACTATCGGTTTCCCA	191	1.467	0.9983
*ABC transporter*	AGCATGATCAAACGGAAGCC/GCTGGCAGGAATAGAAAGGC	159	1.148	0.997

### Expression levels of candidate reference genes

**Fig 2:**
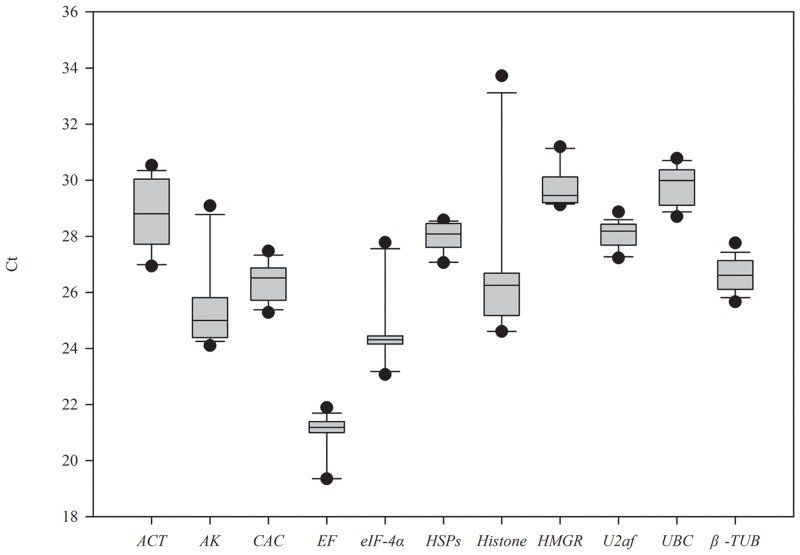
Cycle threshold (Ct) values of the candidate reference genes across the experimental samples. Box-plot graph of Ct value shows the median values as lines across the box. Lower and upper boxes indicate the 25% to 75%. Whiskers represent the maximum and minimum values.

The eleven candidates displayed a relatively wide variation of transcript levels according to average Ct values ranged from 19.35 (*EF*) to 31.19(*HMGR*) (Fig 2), and most of them were in the range of 24–29. The most abundant candidate gene was *EF* and the least one was *UBC*. The expression stability of the candidate genes could also be exhibited by the coefficient of variance (CV) values. The least variation was *U2af* with CV value of 1.69%, while the most variable one was *Histone3* with CV value of 10.62%.

### Expression stability analysis of candidate genes

**Fig 3:**
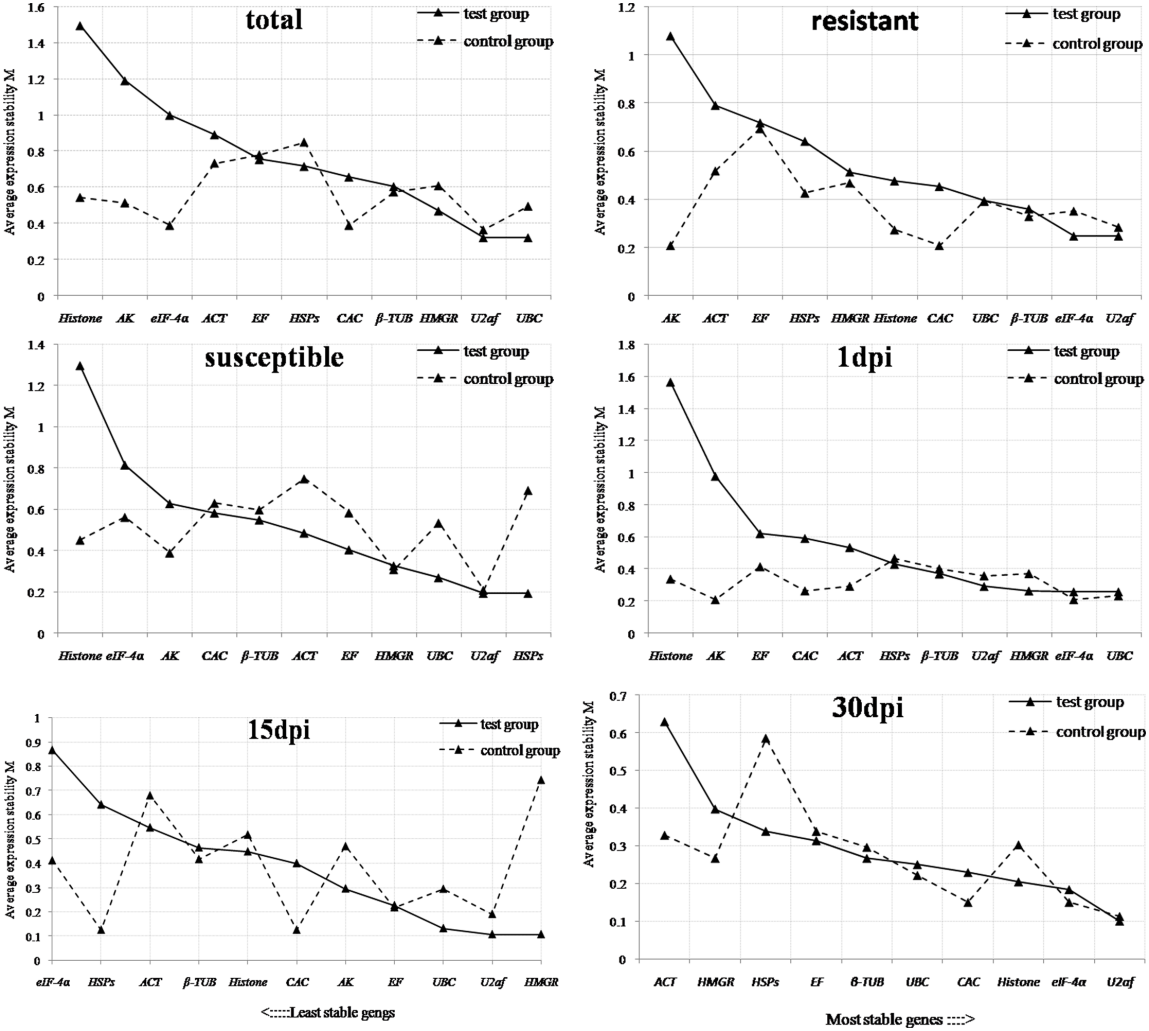
Gene expression stability (M) of candidate genes calculated by geNorm. Ranking of the gene expression stability performed in all the samples, resistant samples, susceptible samples, 1dpi samples, 15dpi samples and 30dpi samples. The least stable genes are on the left and the most stable genes on the right.

**Fig 4:**
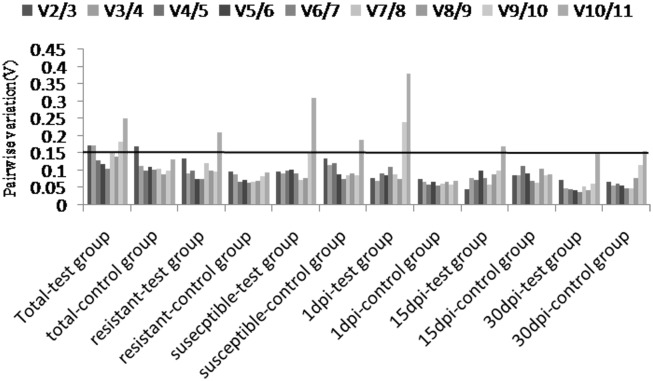
Pairwise variation (V) calculated by geNorm to determine the optimal number of reference genes. The average pairwise variation (Vn/Vn+1) was analyzed to measure the effect of candidate genes on the RT-PCR normalization for all samples.

1) geNorm analysis: On the basis of the pairwise variation between candidate genes, the expression stability value (M) was calculated by geNorm software for all reference genes (Fig 3). The lowest M value indicates the reference gene with the highest stability, whereas the highest M indicates the gene with lowest stability [28]. Expression stability was analyzed in geNorm method across different samples including: 1) total samples, 2) resistant samples, 3) susceptible samples, 4) 1dpi samples, 5) 15dpi samples and 6) 30dpi samples. The ranking order of the genes was in accordance with the average expression stability values. All of 11 candidate reference genes in the study revealed high stability, and M values were less than the geNorm cutoff of 1.6. Therefore, the reference genes with the highest stability could be identical in total samples. In this study, all of tested samples were taken into account and *U2af* and *UBC* had the lowest expression stability, and the highest one was *Histone*. This indicated that the genes with the highest expression stability were *U2af* and *UBC* and the least one was *Histone* among these 11 candidate reference genes. The genes in control groups exhibited a low similarity with the test ones except for *U2af*, *β-TUB* and *EF*. The optimal number of reference genes could be calculated by geNorm used for qRT-PCR and the additional reference genes cannot increase analysis accuracy. The pairwise variation (Fig 4) based on the sequential normalization factors such as NF_n_ and NF_n+1_ showed that V_2/3_ value (0.173) and V_3/4_ value (0.172) were higher than the admitted cutoff value, and the V_4/5_ value was 0.13 (below 0.15). The V_2/3_ value was 0.171 for total samples in the control group. These results indicated that four candidate reference genes would be adequate for the normalization of gene expression in all samples of *P*. *massoniana* subjected to different treatments.

2) NormFinder analysis: The variation of expression stability of reference genes could also be calculated by NormFinder method. The stably expressed candidate reference genes usually manifest the low expression stability. Table 2 showed the stability values for all candidate reference genes through NormFinder analysis. The stability ranking of the reference genes through NormFinder analysis was different from geNorm analysis. The four genes with the highest stability in total samples were *CAC* (0.228), *β-TUB* (0.228), *U2af* (0.488), and *UBC* (0.671). *AK* and *Histone* genes were the reference genes with the lowest genes under both geNorm and NormFinder methods. Meanwhile, the ranking of eleven candidate genes was difference under all kinds of treatments, which was similar with the geNorm method.

**Table 2:** Ranking of candidate reference genes in order according to their expression stability calculated by NormFinder.

Total		Resistant		Susceptible		1dpi		15dpi		30dpi	
ranking	Stability value	ranking	Stability value	ranking	Stability value	ranking	Stability value	ranking	Stability value	ranking	Stability value
*CAC*	0.228	*U2af*	0.136	*CAC*	0.215	*CAC*	0.075	*AK*	0.158	*eIF-4*	0.089
*β-TUB*	0.228	*eIF-4*	0.171	*β-TUB*	0.278	*EF*	0.095	*CAC*	0.161	*Histone*	0.089
*U2af*	0.488	*β-TUB*	0.185	*U2af*	0.333	*ACT*	0.096	*β-TUB*	0.195	*U2af*	0.17
*UBC*	0.671	*CAC*	0.319	*EF*	0.366	*HSPs*	0.147	*Histone*	0.232	*CAC*	0.183
*HSPs*	0.694	*UBC*	0.493	*HMGR*	0.427	*β-TUB*	0.147	*EF*	0.391	*AK*	0.184
*EF*	0.721	*Histone*	0.533	*AK*	0.427	*HMGR*	0.219	*HMGR*	0.459	*HSPs*	0.253
*HMGR*	0.763	*HMGR*	0.704	*HSPs*	0.474	*eIF-4*	0.266	*U2af*	0.462	*EF*	0.261
*ACT*	1.069	*ACT*	0.835	*UBC*	0.541	*UBC*	0.477	*UBC*	0.501	*UBC*	0.347
*eIF-4*	1.217	*HSPs*	0.846	*ACT*	0.604	*U2af*	0.591	*ACT*	0.66	*β-TUB*	0.349
*AK*	1.872	*EF*	1.02	*eIF-4*	1.682	*AK*	2.758	*HSPs*	1.161	*HMGR*	0.732
*Histone*	2.734	*AK*	2.318	*Histone*	3.413	*Histone*	4.183	*eIF-4*	1.862	*ACT*	1.656

3) BestKeeper analysis: The expression stability of candidate genes was ranked according to the coefficient of correlation (r) by the assessment of standard deviation (SD) and the percentage covariance (CV) could be estimated by BestKeeper method (Table 3). In this study, the results from BestKeeper indicated that the highest correlation coefficients were *CAC* (r = 0.911) and *β-TUB*(r = 0.824) with *p*-value of 0.001 across total samples, while *AK* with the r-value of 0.576 and *HSPs* with 0.783 had the lowest *p* (0.001). Due to the discrepant statistical algorithms, the *Histone* and *eIF-4α* with the lowest stability in geNorm and NormFinder analysis were obtained to reveal the high coefficients of correlation of 0.772 and 0.596 with BestKeeper method. Based on the three combinatorial methods, *U2af*, *β-TUB* and *CAC* were the candidate reference genes with the highest stability and *Histone* was the candidate reference gene with the lowest stability in clones of *P*. *massoniana* subjected to different treatments.

**Table 3:** Statistical results by BestKeeper program for eleven selected genes based on Ct values.

Rank	Gene name	r	*p*-value	SD	CV(%)
1	*CAC*	0.911	0.001	0.58	2.2
2	*β-TUB*	0.824	0.001	0.51	1.93
3	*Histone*	0.772	0.001	2	7.41
4	*eIF-4*	0.596	0.009	0.93	3.78
5	*ACT*	0.58	0.012	1.08	3.77
6	*EF*	0.41	0.091	0.56	2.66
7	*UBC*	0.279	0.262	0.58	1.94
8	*U2af*	0.263	0.291	0.39	1.39
9	*HMGR*	0.189	0.451	0.56	1.89
10	*AK*	0.001	0.576	1.18	4.61
11	*HSPs*	0.001	0.783	0.4	1.41

### Validation of reference genes

**Fig 5:**
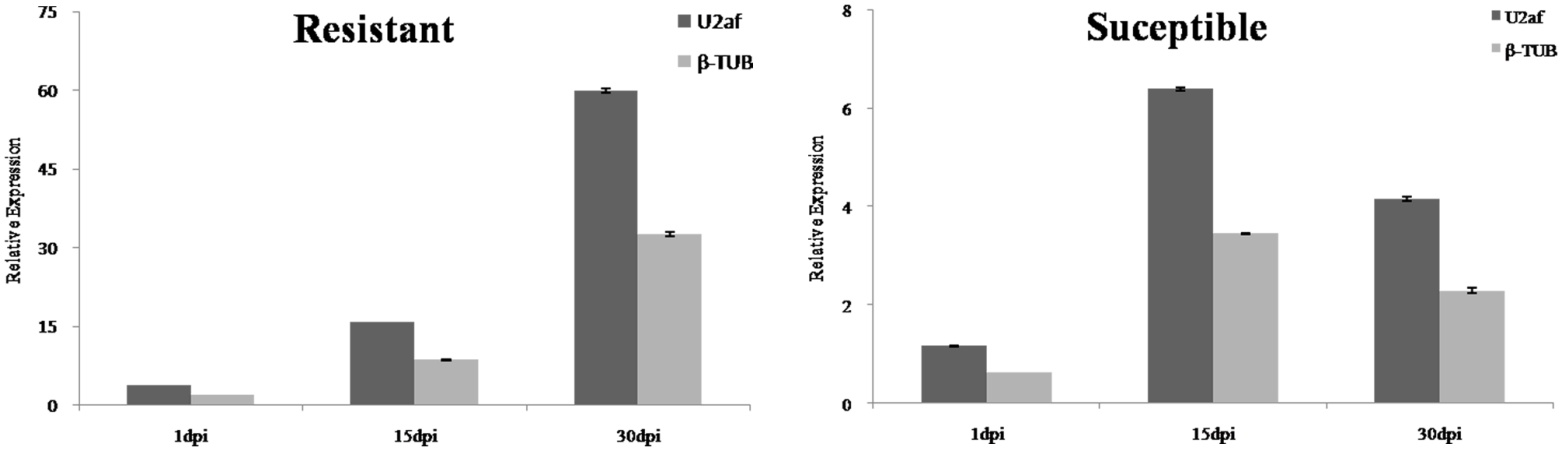
The relative expression of ABC transportor using *U2af* and *β-TUB* as internal controls in both resistant and susceptible clones for three stages post inoculation.

In order to validate the reference genes selected by geNorm, NormFinder and BestKeeper approaches, the relative expression of ATP-binding cassette transporter (ABC transporter) were conducted in resistant and susceptible clones at different stages post inoculation. The selected two genes (*U2af* and *β-TUB*) with higher stability were used for normalizing the qRT-PCR program for the target gene. ABC transporter, closely correlated with the defense mechanisms in pines, could transport constitutive and induced resin to the damaged place. The expression level of ABC transporter is essential when the tree is invaded by PWN. The relative expression of ABC transporter normalized by *U2af* and *β-TUB* exhibited similar trends in three stages (Fig 5). Its transcript revealed continuous increase since the resistant clone was inoculated with nematode. However, the susceptible clone showed a rise-fall trend and its transcript was too low to withstand pine wood nematode on the 30dpi.

## Discussion

In order to improve the resistance of *P*. *massoniana* against PWD, the new transcriptiomics approaches have become the focus of attention [41, 42]. It could be persuasive that the progress of dynamic change is reflected by gene expression analysis of *P*. *massoniana*. The qRT-PCR is a crucial methodology in gene expression analysis. Although many papers on gene expression have used *actin*, *GAPDH*, *EF*, *SAND* or 18s *rRNA* as the reference genes [18, 21, 23], *P*. *massoniana* is not involved in these studies. Additionally, several common reference genes have also been identified, and the significant difference in the expression stability is observed in different plants or samples [19, 34, 36]. Although some of reference genes revealed a low CV value among total samples, it will be essential to implement further studies for evaluating the genes with high stability that can be used in the normalization of gene expression under different experimental treatments. Therefore, the selection of ideal reference genes for expression analysis is highly desired.

In this study, three Excel-based approaches such as geNorm, NormFinder and BestKeeper were used to evaluate the expression stability of candidate reference genes. The cDNA was isolated from resistant and susceptible *P*. *massoniana* during three durations post nematode inoculation: 1dpi, 15 dpi and 30dpi, respectively. The 1dpi was the first day post inoculation; 15dpi was the day that needles began to be flavescent and wilted and the 30dpiwas the day of the tree death. Although there were distinguished features among statistical algorithms and analysis strategies, three approaches showed the same results that *U2af* and *β-TUB* were the candidate genes with the highest stability and *Histone* was the candidate gene with the lowest stability in clones of *P*. *massoniana* subjected to different treatments. *HSPs* and *EF* genes revealed unstable expression both in geNorm and NormFinder analysis but they present relative stability in BestKeeper. On the contrary, *UBC* and *CAC* genes revealed the stable expression by geNorm and NormFinder while the results from BestKeeper showed unstable expression. The incompatibility in stability ranking of candidate genes post inoculation for different days indicated that the expression levels of selected genes might be regulated by *P*. *massoniana* in response to PWN. These incongruent results of the selected reference genes demonstrated that geNorm and NormFinder could not establish the expression stability, thus, BestKeeper approach was also suitable to increase the confidence of the gene expression stability [19]. Except several statistical methods leading to the discrepant stability, another distinguish was emerged in the expression of candidate genes in resistant and susceptible samples by identical approach, which suggested that various reactions could be detected in clones of *P*. *massoniana* in response to PWN invasion [15]. In previous reports, *EF* and *ACT* were usually applied for normalizing the quantification of gene expression in other plants [43, 44], while *ACT* was estimated as an unstable reference gene in present study. *U2af* and *β-TUB* were recommended to be the best reference genes to normalize qRT-PCR results in *P*. *massoniana* post inoculation. The results were consistent with the study of *U2af* gene in *P*. *taeda* [18] and *β-TUB* gene in *Sedum alfredii* [43].

Genes usually viewed as housekeeping genes including *actin*, *tubulin*, splicing factor *U2af* and *ubiquitin* have been widely employed to normalize gene expression, whereas, the misinterpretation might turn up when these housekeeping genes were used discretionarily. As a common stable reference gene in many species including *Pinus pinaster* Ait. [19, 23], *Pinus pinea* Lamb. [16], *Pinus taeda* Lamb. [17–18], *eIF-4* was used for the normalization in the process. However, it was inappropriate to normalize the qRT-PCR program in the present study. It showed highly expression abundance but the expression was unstable in different treatments post inoculation. As an important factor in precursor mRNA splicing, the stable *U2af* has been corroborated in *P*. *taeda* [18] and switch grass [45] so that it can be stably expressed in *P*. *massoniana* post inoculation and could be regarded as the reference gene. According to the unacceptable expression stability, *Histone* and *AK* were predicated as the genes with the lowest stability in three approaches simultaneously, and excluded from the reference genes in inoculated *P*. *massoniana* studies. Given these observations, the evaluation process is necessary when both *Histone* and *AK* are considered as the reference genes for normalizing gene expression.

The optimal reference genes in *P*. *massoniana* is the following four genes, *U2af*, *β-TUB*, *CAC* and *UBC*. Each one displayed a low stability value in geNorm approach (M<1.0). The analysis concurrently indicated that the stability of other candidate genes reduced gradually and they would not be prior to use in the gene expression study. In different expression profiles, the difference was indistinctive when two reference genes were used for the normalization compared to three or four genes [16]. These subtle differences will not be integrating in most of experiments, which can demonstrate that the addition of a third or fourth gene for increasing the accuracy may not be essential [46]. The conclusion was drawn that two genes such as *U2af* and *β-TUB* can be competent to quantify the transcript in gene expression studies.

The expression levels of ABC transporter genes in different resistant *P*. *massoniana* clones and different stages post inoculation were used to validate the reliability of *U2af* and *β-TUB* as reference genes. The expression profile of ABC transporter with two internal controls revealed similar trends, respectively. However, the tendency of ABC transporter transcript was significant difference between resistant and susceptible clones. The resistant clones could continually transport resin to restrain the invasion of reproducing nematodes; on the contrary, the susceptible ones were killed by the constantly reproduced nematode. In the susceptible trees, the constitutive and induced resin revealed the inhibition for the migration and reproduction of nematodes from 1dpi to 15dpi. The ABC transporter could lead to the continuous expression. With the increase of the population, plentiful nematodes can invade into xylem to result in the damage of resin canal. Thus, the resin cannot be transported and the defense mechanism can be interrupted. These may be the reason for the reduction of ABC transporter between 15dpi and 30dpi. These results were consistent with the study of *P*. *thunbergii* infected with nematode [47].

In the future work, the selected genes will be further used for gene expression analysis in cultivars with high resin yield and compare with the low ones in order to illuminate the relationship between resin and resistance to pine wilt disease. According to qRT-PCR analysis, the expression profiles of functional genes associated with resin biosynthesis and PWN resistance can be informative to understand the nature of resistant/susceptible clones. This work will facilitate to the understanding of molecular events from a series of gene expression analysis in *P*. *massoniana* post nematode inoculation.

## Materials and Methods

### Plant materials and treatments

A resistant tree from the variety ‘Xiuning 5’, the variety of *P*. *massoniana* with the highest resistance was selected as a part of breeding project, and was planted in germplasm nursery of the Anhui Academy of Forestry, Anhui Province, China. A susceptible tree from the variety ‘Huangshan 1’ was selected as a plus-tree for the control group was planted in the Anhui Academy of Forestry. Both clones used in the present study were grafts obtained from the original trees at the Anhui Academy of Forestry in 2010. The grafted saplings were cultivated for 4 years.

Inoculation with a highly virulent isolate (Guangzhu-3B) of PWN used for pine wilt disease resistance breeding projects [14] was conducted on July 20, 2014. In three susceptible and resistant clones, we cut the tree bark slantly with a knife for approximately 3cm in depth until it was inserted into the xylem, which was similar with the biting situation of *Monochamus alternatus* Hope. In addition, 20μL of sterile water with the suspension of 10000 nematodes was injected into each slot homogeneously. Non-inoculated samples were injected with sterile water without nematodes into the cut of the stem in additional three susceptible clones and three resistant clones.

Stem segments at 1–3cm above the inoculation point were sampled from inoculated trees and non-inoculated trees at 1, 15 and 30 days post inoculation (dpi). Each treatment was conducted in triplicate. The segments with 2cm in length were frozen in liquid nitrogen immediately, and then stored at -80°C until RNA extraction.

### Total RNA isolation and cDNA synthesis

Total RNA was isolated using the EASY spin Plus Plant RNA Kit (Aidlab Biotechnologies Co., Ltd, Beijing, China) according to the manufacturer’s instructions. The concentration and quality of RNA samples were determined using NanoDrop 2000 spectrophotometer (NanoDrop Technologies, Wilmington, DE, USA). The quality of RNA was also assessed by 2% agarose gel electrophoresis. Only DNA with absorbance ratio of A260/A280 in the range of 1.8–2.2 and A260/A230 at above 1.0 could be used for cDNA synthesis.

First-strand cDNA was synthesized from approximately 1μg of total RNA in a total volume of 20μL of reaction mixture through TURE script 1^st^ Strand cDNA Synthesis Kit (Aidlab Biotechnologies Co., Ltd, Beijing, China) following the manufacturer’s protocol. The cDNA products were diluted to a final volume of 800μL to be used in the quantitative real-time RT-PCR (qRT-PCR).

### Selection of candidate reference genes and primer design

A total of eleven genes commonly used as reference genes in different plant species were selected for the evaluation as the control genes in *P*. *massonsana*, which was confirmed from published data in other plants [5, 19, 43, 45, 48]. Primer3 program (http://primer3.ut.ee) was used to design the primer pairs for all those genes with the optimal T_m_ in the range of 58–62°C, product size ranging from 120 to 220bp, primer length between 19 and 21 nucleotides and the GC content of 40–55%. The products of qRT-PCR with 2% agarose gel electrophoresis and melting curve analysis were analyzed in order to verify the single PCR product amplified for each set of primers.

### qRT-PCR

The qRT-PCR amplification was conducted in 96-well plates using a 7300 Real Time PCR System (Applied Biosystems, CA, USA) with 2×SYBR Green qPCR Mix (Aidlab Biotechnologies Co., Ltd, Beijing, China) according to the manufacturer’s protocol. Each PCR reaction was completed in a volume of 20μL containing 2μL of cDNA, 10μL of 2×SYBR qPCR Mix, 0.4μL of 10μM forward primer and reverse primer, 7μL of double distilled water and0.2μL of ROX reference dye. PCR reaction was conducted through following program including the pre-denaturation at 95°C for 30s and 40 cycles of the standard thermal amplification cycling with denaturation at 95°C for 15s, annealing at 58°C for 30s and extension at 72°C for 30s. The specificity of PCR reaction was verified by melting curve analysis of the amplified product for each sample. Each qRT-PCR reaction was conducted in triplicate (technical replicates) for three individual plants (biological replicates).

### Analysis of gene expression stability

Standard curves were used to calculate the gene-specific PCR efficiency from 10-fold series dilution of the mixed cDNA template for each primer pair. The correlation coefficients (*R*^*2*^) and slope values were acquired from the standard curve, and PCR amplification efficiencies (*E*) were calculated according to the equation *E* = (10^−1/slope^-1)×100%.

The geNorm, NormFinder and BestKeeper approaches were implemented to analyze the expression stability of the candidate reference genes. The Ct values of all samples were collected to select stably expressed genes. The stability is calculated by Ct data with BestKeeper, and these Ct values are converted to relative quantity following the Δ-Ct method to implement geNorm and NormFinder analysis. The geNorm software is used to calculate the expression stability value (M) for reference genes based on the mean pairwise variation between all tested genes (http://medgen.ugent.be/~jvdesomp/genorm/), and NormFinder is used to rank the expression stability of reference genes according to their intra and inter expression variations (http://www.mdl.dk/publicationsnormfinder.htm). The BestKeeper can be used an index for the evaluation of expression stability. It is calculated by standard deviation (SD) and percentage covariance (CV) (http://www.genequantification.de/bestkeeper.html).

## Supporting Information

S1 Fig The relative expression of ABC transporter compared with 11 primers. (DOCX)

S1 Table Functional annotation of the Pinus Massonaia transctiptome. (XLSX)

S2 Table Primers information of the 11 genes. (XLSX)
