# Supplementary material for: Selection of Reference Genes for Real-Time Quantitative PCR in Pinus massoniana Post Nematode Inoculation
Source: PLoS One. 2016 Jan 22;11(1):e0147224. doi: 10.1371/journal.pone.0147224 (PMC4723224; doi:10.1371/journal.pone.0147224)
Supplement: S1 Fig — (DOCX) [file pone.0147224.s001.docx]

**Fig. The relative expression of ABC transporter compared with 11 primers.**
